# Ferulic acid suppresses the inflammation and apoptosis in Kawasaki disease through activating the AMPK/mTOR/NF-κB pathway

**DOI:** 10.3389/fphar.2024.1420602

**Published:** 2024-08-29

**Authors:** Huilan Wu, Yijia Wang, Pingping Tan, Yuqing Ran, Yuting Guan, Songwei Qian, Xing Feng, Yalan Jiang, Yongmiao Peng, Ke Sheng, Haitao Xi, Weiping Ji, Xiaoling Guo

**Affiliations:** ^1^ Basic Medical Research Center, The Second Affiliated Hospital and Yuying Children’s Hospital of Wenzhou Medical University, Wenzhou, Zhejiang, China; ^2^ Department of General Surgery, The Quzhou Affiliated Hospital of Wenzhou Medical University, Quzhou People’s Hospital, Quzhou, Zhejiang, China; ^3^ Reproductive Medicine Center, Department of Obstetrics and Gynecology, The Second Affiliated Hospital of Wenzhou Medical University, Wenzhou, Zhejiang, China; ^4^ Department of General Surgery, The Second Affiliated Hospital and Yuying Children’s Hospital of Wenzhou Medical University, Wenzhou, Zhejiang, China; ^5^ Scientific Research Department, The Second Affiliated Hospital and Yuying Children’s Hospital of Wenzhou Medical University, Wenzhou, Zhejiang, China; ^6^ Key Laboratory of Structural Malformations in Children of Zhejiang Province, The Second Affiliated Hospital and Yuying Children’s Hospital of Wenzhou Medical University, Wenzhou, Zhejiang, China

**Keywords:** kawasaki disease (KD), ferulic acid (FA), coronary artery, vasculitis, inflammation, apoptosis

## Abstract

**Background:**

Kawasaki disease (KD) is a self-limiting and acute systemic vasculitis of unknown etiology, mainly affecting children. Ferulic acid (FA), a natural phenolic substance, has multiple pharmacological properties, including anti-inflammatory, anti-apoptosis, and anti-fibrosis, and so on. So far, the protective effects of FA on KD have not been explored.

**Methods:**

In this study, we established *Candida* albicans water soluble fraction (CAWS)-induced mouse coronary artery vasculitis of KD model and the tumor necrosis factor α (TNF-α)-induced human umbilical vein endothelial cells (HUVECs) injury model to investigate the anti-inflammatory and anti-apoptosis effects of FA on KD, and try to elucidate the underlying mechanism.

**Results:**

Our *in vivo* results demonstrated that FA exerted anti-inflammatory effects on KD by inhibiting the infiltration of CD45-positive leukocytes and fibrosis around the coronary artery. Additionally, FA downregulated the levels of inflammatory and chemotactic cytokines, alleviated splenomegaly, and exhibited anti-apoptotic effects on KD by reducing TUNEL-positive cells, downregulating BAX expression, and upregulating BCL-2 expression. In addition, Our *in vitro* findings showed that FA could effectively inhibit TNF-α-induced HUVEC inflammation like NF-κB inhibitor QNZ by downregulating the expression of pro-inflammatory cytokines as well as attenuated TNF-α-induced HUVEC apoptosis by reducing apoptotic cell numbers and the BAX/BCL-2 ratio, which could be reversed by the AMPK inhibitor compound c (CC). The further mechanistic study demonstrated that FA could restrain vascular endothelial cell inflammation and apoptosis in KD through activating the AMPK/mTOR/NF-κB pathway. However, FA alone is hard to completely restore KD into normal condition.

**Conclusion:**

In conclusion, FA has potential protective effects on KD, suggesting its promising role as an adjuvant for KD therapy in the future.

## Introduction

Kawasaki disease (KD) is an acute, self-limiting fever disease characterized by systemic vasculitis, primarily affecting the coronary arteries and occurring in children under 5 years old (Newburger et al., 2016). The most significant complication of KD is the occurrence of coronary aneurysms caused by coronary artery injury. Therefore, early detection, diagnosis, and treatment are particularly important for KD. To date, KD has been the main cause of acquired heart disease among children in developed countries (Harnden et al., 2009). Currently, KD is mainly treated by timely intravenous immunoglobulin (IVIG), but more than 20% of KD patients are tolerant to IVIG and need adjuvant drug therapy (McCrindle et al., 2017). In the Japanese population, corticosteroid adjuvant therapy has a certain effect on high-risk KD patients with coronary complications (Shulman and Rowley, 2015). Therefore, it is imperative for us to explore alternative therapeutic agents that may have potential protective effects on KD.

The application of KD mouse model with vasculitis has greatly improved our understanding of KD pathology. The classical methods of KD mouse modeling mainly include *Lactobacillus* casei cell wall component (LCWE)-induced KD mouse model, *Candida* albicans water-soluble fraction (CAWS)-induced KD mouse model (Lehman et al., 1985; Murata, 1979), and the Nod1 ligand-mediated KD mouse model (Noval Rivas and Arditi, 2020). *Candida* albicans is a harmless symbiotic fungus commonly found in the human gastrointestinal tract, and CAWS consists of polysaccharides such as α-mannans and β-glucans within the yeast cell wall (Ohno, 2003). Mice were intraperitoneally injected with CAWS for consecutive 5 days, which could induce a typical KD clinical phenotype (Noval Rivas and Arditi, 2020). In addition, serum TNF-α level in KD patient is significantly increased, and TNF-α plays a promoting role in inducing coronary artery inflammation and the occurrence of coronary artery aneurysms (Jiang et al., 2016). In this study, we would utilize CAWS to induce coronary arteritis to establish KD mouse model *in vivo*, and employ TNF-α to induce human umbilical vein endothelial cell (HUVEC) injury to establish KD cell model *in vitro*.

Necrotizing arteritis, subacute chronic vasculitis, and intraluminal myofibroblast proliferation are the three main pathological manifestations of KD (Orenstein et al., 2012). Histopathological analysis revealed significant destruction and proliferation of all three layers of coronary artery wall in KD patients, and vascular endothelial cells were stripped and exposed to a large number of infiltrating inflammatory cells, resulting in endothelial cell apoptosis and dysfunction (Gavin et al., 2003). Ferulic acid (FA), a phenolic substance, is widely found in plants and has various pharmacological effects, such as anti-inflammatory, anti-apoptosis, anti-fibrosis, and so on (Chen et al., 2019; Rehman et al., 2019; Zhao et al., 2020). The function of FA is highly matched to the pathological features of KD, including inflammation, apoptosis, and fibrosis. Therefore, we hypothesized that FA might possess therapeutic potential for KD. So far, there have been no study to report the protective effects of FA on KD. In our study, we would investigate the protective roles of FA on coronary artery injury in CAWS-induced KD mouse model *in vivo* and endothelial cell injury in TNF-α-induced KD HUVEC model *in vitro*, attempting to explore the underlying mechanism.

## Materials and methods

### Reagents

The powder of ferulic acid (FA, Aladdin, Shanghai, China) was dissolved in 0.9% saline solution containing 0.3% carboxymethyl cellulose to prepare the stock concentration, and fresh FA with working concentration was prepared within 1 h before the experiments. For *in vivo* experiments, the working concentrations of FA were set at a low dose of 40 mg/kg (L-FA) and a high dose of 80 mg/kg (H-FA), which were based on a previous study (Liu et al., 2017). For *in vitro* experiments, the working concentration of FA was determined using the subsequent CCK-8 assay. The *candida* albicans water-soluble fraction (CAWS) extracted from *Candida* albicans NBRC1385 was prepared using the method described in the literature (Stock et al., 2016). The harvested CAWS was used to induce KD mouse model *in vivo*.

### Animals and experimental design

The four-week-old C57BL/6J male mice (n = 120) were purchased from Weitong Lihua Company (Hangzhou, China) and placed in the standard mouse cages with a 12 h dark/light cycle, the temperature of 23°C ± 2°C, and the humidity of 50% ± 5%. Water and food were accessed *ad libitum*. The animal experiment of this study was approved by the Wenzhou Medical University’s Animal Care and Use Committee, and conducted in accordance with the Guide for the Care and Use of Laboratory Animals. The mice were randomly divided into four groups: control group (n = 10), CAWS group (n = 10), CAWS + L-FA group (n = 10), and CAWS + H-FA group (n = 10). The independent biological experiment *in vivo* for each group were conducted 3 times. A daily intraperitoneal injection of 4 mg/kg CAWS was administered for five consecutive days to induce mouse KD model. Then, the CAWS-induced mice were oral gavage of either 40 mg/kg FA (L-FA) or 80 mg/kg FA (H-FA) daily for two consecutive weeks. Mice in control group received an oral gavage of equal volume 0.9% saline with 0.3% carboxymethyl cellulose. On day 28 after CAWS injection, the mice in each group were euthanized by overanesthesia, and their heart and spleen tissues were collected for further experiments.

### Human umbilical vein endothelial cell (HUVECs) culture and treatment

HUVECs (ATCC, VA, United States) were cultured in high-glucose DMEM medium with 10% fetal bovine serum (FBS, Gibco, NY, United States) and 1% penicillin/streptomycin (P/S) at a 37°C, 5% CO_2_ incubator, and the medium was refreshed every other day. TNF-α (PeproTech, NJ, United States) was used to induce HUVEC KD model, and the optimum working concentration of TNF-α was determined by CCK-8 experiments. HUVECs were divided into different treatment groups: control group, TNF-α group, TNF-α+FA group, and TNF-α + QNZ group. We also set another groups such as control group, TNF-α group, TNF-α + FA group, and TNF-α + FA + CC group. HUVECs were pre-treated with TNF-α (1 μg/mL) and then treated with FA (20 μM) or a mixture of FA (20 μM) and the p-NF-κB inhibitor QNZ (10 nM, Selleck, TN, United States) or FA (20 μM) and the AMPK inhibitor compound c (CC, 10 μM, MedChem Express Biotechnology, NJ, United States) for another 24 h. The working concentrations of QNZ and CC were based on a previous report (Jian et al., 2019; Wang et al., 2024).

### Real-time quantitative polymerase chain reaction (RT-qPCR)

Total RNAs were extracted from the aortic root and proximal coronary artery regions of mouse heart tissues or HUVECs in each group using Trizol reagent (Invitrogen, CA, United States), and the concentration of RNAs was detected by an ultra microspectrophotometer (Nanodrop 2000, Thermo, MA, United States). The complementary DNA (cDNA) templates were then generated from the reverse transcription of isolated RNAs using HiScript II Q RT SuperMix (Vazyme Biotech, Nanjing, China). The harvested cDNAs were subjected to RT-qPCR using ChamQ Universal SYBR qPCR Master Mix (Vazyme Biotech, Nanjing, China). The reaction program was 30 s at 95°C (pre-denaturation), 10 s at 95°C (denaturation), and 30 s at 60°C (40 cycles). Subsequently, the reaction system was slowly heated from 60°C to 95°C to establish the melting curve after the amplification. *Gapdh* transcript served as the endogenous control. Finally, the relative changes in gene expressions were calculated using the 2^−ΔΔCT^ method. The information of primers was shown in Table 1.

**TABLE 1:** Primers.

Primer information for mouse			
Primer Symbol	Gene name	Primer direction	Sequences (5’to 3’)
IL-1β	Interleukin 1 beta	Forward	TGA​AAT​GCC​ACC​TTT​TGA​CAG​TG
		Reverse	ATG​TGC​TGC​TGC​GAG​ATT​TG
IL-6	Interleukin 6	Forward	AAT​TTC​CTC​TGG​TCT​TCT​GGA​GT
		Reverse	TCT​GTG​ACT​CCA​GCT​TAT​CTC​TTG
TNF-α	Tumour necrosis factor alpha	Forward	ACC​GTC​AGC​CGA​TTT​GCT​AT
		Reverse	CTC​CAA​AGT​AGA​CCT​GCC​CG
GAPDH	Glyceraldehyde-3-phosphate dehydrogenase	Forward	GGC​AAA​TTC​AAC​GGC​ACA​GT
		Reverse	CCT​TTT​GGC​TCC​ACC​CTT​CA
CXCL5	chemokine (C-X-C motif) ligand 5	Forward	CCC​TTC​CTC​AGT​CAT​AGC​CG
		Reverse	CTT​CCA​CCG​TAG​GGC​ACT​G
CXCL10	chemokine (C-X-C motif) ligand 10	Forward	CTC​ATC​CTG​CTG​GGT​CTG​AG
		Reverse	CAA​CAC​GTG​GGC​AGG​ATA​GG
CCL2	C-C motif chemokine ligand 2	Forward	GTC​TGT​GCT​GAC​CCC​AAG​AA
		Reverse	GAC​CTT​AGG​GCA​GAT​GCA​GTT
CCL7	C-C motif chemokine ligand 7	Forward	CTT​TCA​GCA​TCC​AAG​TGT​GGG
		Reverse	CTC​GAC​CCA​CTT​CTG​ATG​GG
CCL17	C-C motif chemokine ligand 17	Forward	AGG​GAG​CCA​TTC​CCC​TTA​GA
		Reverse	CTC​TTG​TTG​TTG​GGG​TCC​GA

Primer information for human			
Primer Symbol	Gene name	Primer direction	Sequences (5’to 3’)
GAPDH	Glyceraldehyde-3-phosphate dehydrogenase	Forward	GGA​GCG​AGA​TCC​CTC​CAA​AAT
		Reverse	GGC​TGT​TGT​CAT​ACT​TCT​CAT​GG
IL-1β	Interleukin 1 beta	Forward	AGC​CAT​GGC​AGA​AGT​ACC​TG
		Reverse	CCT​GGA​AGG​AGC​ACT​TCA​TCT
IL-6	Interleukin 6	Forward	CCT​TCG​GTC​CAG​TTG​CCT​TC
		Reverse	GAT​GCC​GTC​GAG​GAT​GTA​CC
CXCL10	chemokine (C-X-C motif) ligand 10	Forward	CCT​GCA​AGC​CAA​TTT​TGT​CCA
		Reverse	AGA​CCT​TTC​CTT​GCT​AAC​TGC​T

### Western blot analysis

Total proteins were extracted from the aortic root and proximal coronary artery regions of mouse heart tissues or HUVECs in each group using RIPA lysis buffer containing a mixture of protease inhibitor and phosphatase inhibitor (Beyotime, Shanghai, China). The concentration of protein was detected using the BCA protein detection kit (Beyotime, Shanghai, China), and then proteins were denatured at 100°C for 10 min. The denatured proteins were separated by 12% SDS-PAGE gel and transferred into PVDF membranes (Thermo Fisher, MA, United States). The membranes were blocked with 5% skim milk, and then were incubated overnight at 4°C with the primary antibodies (listed in Table 2). After washing three times, they were incubated with horseradish peroxidase-conjugated secondary antibody (1:1,000, Beyotime, Shanghai, China) at room temperature for 2 h. Protein bands were visualized using ChemiDicTM XRS Imaging System (Bio-Rad, CA, United States), and the density of protein band was quantified using ImageJ software.

**TABLE 2:** Antibodies.

Antibody	Species	Company (catalogue)	Dilution	
			WB	IHC/IF
GAPDH	Rabbit	Affinity (AF7021)	1:10000	ND
CD31	Goat	R& Systems D (#Q08481)	ND	1:200
IL-1β	Mouse	Santa Cruz Biotechnology (sc-52012)	1:1000	ND
IL-6	Mouse	Santa Cruz Biotechnology (sc-32296)	1:1000	ND
CD45	Rabbit	Abcam (ab10558)	1:500	1:100
AMPK	Mouse	Santa Cruz Biotechnology (sc-398861)	1:1000	ND
p-AMPK	Rabbit	Cell Signaling Technology (2535S)	1:1000	ND
mTOR	Mouse	Santa Cruz Biotechnology (sc-517464)	1:1000	ND
p-mTOR	Mouse	Santa Cruz Biotechnology (sc-293133)	1:1000	ND
NF-κB	Mouse	Santa Cruz Biotechnology (sc-8008)	1:1000	ND
p-NF-κB	Mouse	Santa Cruz Biotechnology (sc-166748)	1:1000	ND
BAX	Mouse	Santa Cruz Biotechnology (sc-7480)	1:1000	ND
BCL-2	Mouse	Santa Cruz Biotechnology (sc-7382)	1:1000	ND

ND, Not detected; WB, Western blot; IHC, Immunohistochemistry; IF, Immunofluorescence.

### Hematoxylin and eosin (H&E) staining

The mouse the aortic root and proximal coronary artery regions of mouse heart tissues in each group were fixed with 4% paraformaldehyde, dehydrated with gradient alcohol, and embedded in paraffin. Tissue sections with a thickness of 5 µm were dewaxed, hydrated, and then stained with H&E solution using a Hematoxylin-Eosin staining kit (Solarbio, Beijing, China). After clearing, all sections were sealed with the neutral resin and observed under a pathology scanner (KFBIO, KF-PRO-005, Ningbo, China).

### Masson staining

After dewaxing and hydration, the sections of the aortic root and proximal coronary artery regions of mouse heart tissues in each group were stained with the prepared Weigert iron hematoxylin staining solution, washed with acidic ethanol differentiation solution after differentiation, and Masson bluing solution after bluing for 5 min. Ponceau-fuchsin staining solution was washed with weak Acid working solution after dyeing, and phosphomolybdic Acid solution was washed with weak Acid working solution after differentiation. Finally, the aniline blue dyeing solution was washed with weak Acid working solution.

### Immunofluorescence staining

The HUVECs in each group were fixed in 4% paraformaldehyde for 15 min and blocked in a solution of 3% bovine serum albumin containing 0.3% Triton X-100 (Sigma, NM, United States) at 4°C for 1 h according the previous report (Xing et al., 2024a). They were then incubated with primary antibodies (listed in Table 2) overnight at 4°C. After washing three times with PBS, they were treated with Cy3-conjugated secondary antibody at room temperature for 2 h in the dark. Finally, the cells were sealed using a sealing solution containing DAPI, and images were captured using an integrated fluorescence microscopy imaging system (Keyence Corporation, Osaka, Japan).

### Immunohistochemical staining

The paraffin sections of the aortic root and proximal coronary artery regions of mouse heart tissues in each group were dewaxed and hydrated. The entire section was covered with a peroxidase blocker and then stored at room temperature for 20 min. The sections were then rinsed with PBS and underwent antigens retrieval under high pressure in 10 mM citrate buffer (pH 6.0). The sections were then blocked with 10% goat serum at room temperature for 1 h. These sections were then incubated with primary antibodies (listed in Table 2) overnight at 4°C. After washing three times with PBS, theses sections were incubated with secondary antibodies (1:50, Beyotime, Shanghai, China) for 2 h at room temperature. The color of section was developed using DAB color development kit (Zsbio, Beijing, China). After staining with hematoxylin, they were dehydrated and transparent, sealed with neutral resin (Xing et al., 2024b), and observed under an optical microscope (Nikon, Tokyo, Japan).

### TUNEL staining

One-step TUNEL apoptosis assay kit (Beyotime, Shanghai, China) was used to assess the apoptosis of the aortic root and proximal coronary artery regions of mouse heart tissues or HUVECs in each group. Briefly, the tissues were dewaxed and treated with 20 μg/mL protease K without DNase at 20°C–37°C for 15–30 min. The cells were fixed with 4% paraformaldehyde for 30 min and then incubated with PBS containing 0.3% Triton X-100 at room temperature for 5 min. The samples of tissues and cells were incubated with TUNEL assay solution at 37°C for 60 min and then stained with DAPI in the dark. Lastly, images were captured under an inverted fluorescence microscope (Leica, Hesse, Germany).

### Flow cytometry of Annexin V and PI assay

The FITC-Annexin V/PI apoptosis detection kit (KeyGEN, Nanjing, China) was used to detect HUVEC apoptosis in each group. The cells were digested with EDTA-free pancreatic trypsin (Gibco, NY, United States), washed twice with pre-cooled PBS, and then resuspended in 1× binding buffer containing 5 µL of FITC Annexin V and 5 µL of PI. After thorough mixing, the suspension was kept at room temperature in the dark for 50 min. Finally, 400 µL of 1× binding buffer was added, and the ratio of cell apoptosis was analyzed by the flow cytometry (Beckman, CA, United States).

### Cell counting Kit-8 (CCK-8)

Cell counting kit-8 (New Cell and Molecular Biotech, Suzhou, China) was used to assess cell viability of HUVECs in each group. Briefly, cells were seeded in 96-well plates and cultured at a 37°C 5% CO_2_ incubator for 24 h. Then, the medium was changed with fresh medium with different concentrations (0.001 ng/mL, 0.01 ng/mL, 0.1 ng/mL, 1 ng/mL, 10 ng/mL, 100 ng/mL, 100 ng/mL, and 1,000 ng/mL) of TNF-α, and cells were incubated for another 24 h. 100 μL of mixture including 90 μL of fresh medium and 10 μL of CCK-8 solution was added to each well and maintained at 37°C for 1 h. The absorbance value at 450 nm was measured using a Microplate Reader (Thermo, MA, United States). The cell viability in each group is calculated as follows: cell viability (%) = [(absorbance of experimental wells - absorbance of blank wells)/(absorbance of control wells - absorbance of blank wells)] × 100%.

### Statistical analysis

All results are expressed as mean ± standard deviation (SD). The letter “n” in figure legends means the number of biological replicates, and the “points” in the graphs means the analyzed sample size. The sample size for *in vitro* (n ≥ 3) and *in vivo* (n ≥ 5) are typical of the field. The statistical significance of the differences was evaluated by GraphPad Prism version 9 (GraphPad Software Inc., CA, United States). The Student’s t-test was used to compare the two groups of data. Multi-group comparisons were performed using one-way analysis of variance (ANOVA), followed by Turkey’s multiple comparisons when comparing more than two groups. A *p*-value less than 0.05 was considered statistically significant.

## Results

### FA reduced inflammation around coronary artery in CAWS-induced mice

**FIGURE 1:**
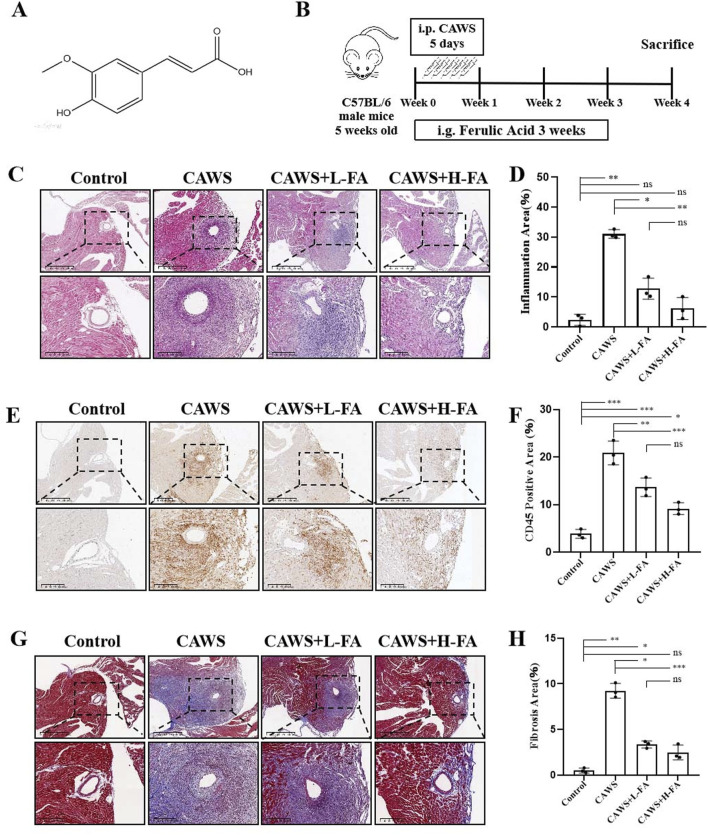
FA alleviated coronary artery inflammation in CAWS-induced KD mice. **(A)** The chemical structure of FA. **(B)** A flow diagram illustrating the experimental procedure for inducing Kawasaki disease (KD) in mice and treating them with FA. **(C)** HE staining of around mouse coronary artery in different groups. Scale bars 625 μm, 200 μm. **(D)** The quantification of HE staining in different groups (n = 3). **(E)** Immunohistochemistry of CD45 in different groups. Scale bars 625 μm, 200 μm. **(F)** The quantification of CD45 immunohistochemistry in different groups (n = 3). **(G)** Mason staining around mouse coronary artery in different groups. Scale bars 400, 200 μm. **(H)** The quantification of Mason staining in different groups (n = 3). Mean ± SD, ^*^ *p* < 0.05, ^**^ *p* < 0.01, ^***^ *p* < 0.001 indicate significant differences, and *ns* > 0.05 means no significance difference.

The compound 3-(4-hydroxy-3-methoxyphenyl)-2-propenoic acid is commonly referred to as Ferulic acid, also known as 4-hydroxy-3-methoxycinnamic acid. Figure 1A illustrates the molecular structure of trans-Ferulic acid, which is more popular used in the cosmetics and pharmaceutical industries than cis-Ferulic acid (Graf, 1992). The diagram depicted the KD modeling in CAWS-induced mice from day 0 to day 5 as well as the drug administration from day 0 to day 21 (Figure 1B). On day 28, the aortic root and proximal coronary artery regions of mouse heart tissues in each group were taken for HE staining to assess the inflammatory infiltration around the coronary artery (Figure 1C). The results of HE staining showed that compared with control group, there was serious inflammatory infiltration around the coronary artery in CAWS group, but either L-FA or H-FA could alleviate this symptom. Although there was no significant difference between control and L-FA or H-FA treatment, the inflammatory infiltration of either CAWS + L-FA or CAWS + H-FA group was more serious than that of control group (Figure 1D). CD45, known as leukocyte common antigen, is one of the important biomarkers in the inflammatory response (Kuai et al., 2022). Immunohistochemical staining of CD45 was performed in each group (Figure 1E). The results showed that there was almost no CD45-positive cells around the coronary artery in control group, but there were numerous CD45-positive cells in CAWS group. The administration of L-FA or H-FA could significantly decrease CD45-positive cells around mouse coronary artery, but the CD45-positive cells in either CAWS + L-FA or CAWS + H-FA group were significantly more than these of control group (Figure 1F). Masson staining was conducted to assess the fibrosis around the coronary artery (Figure 1G). The results of Masson staining are almost consistent with the above trend of HE staining (Figure 1H). These findings suggest that FA can reduce inflammation in mouse coronary arteries of CAWS-induced KD mice, but FA alone is hard to completely restore KD mice into normal condition.

### FA decreased the levels of inflammatory cytokines in CAWS-induced KD mice

**FIGURE 2:**
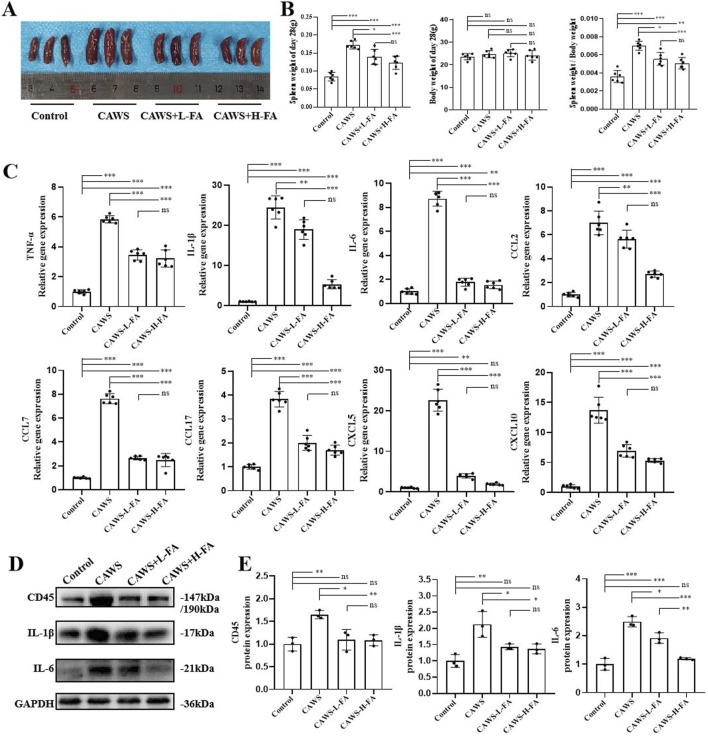
FA decreased inflammation in CAWS-induced KD mice. **(A)** The representative images of mouse spleens in different groups on day 28. **(B)** Spleen weight, body weight, and the ratio of spleen weight/body weight on day 28 in different groups (n = 3). **(C)** The mRNA expression levels of inflammatory cytokines and chemokines in the aortic root and proximal coronary artery regions of mouse heart tissues in each group were evaluated using RT-qPCR (n = 3). **(D)** Western blot analysis was employed to assess the expression of CD45, IL-1β, and IL-6 proteins in the aortic root and proximal coronary artery regions of mouse heart tissues in each group. **(E)** The quantification of western blot in different groups (n = 3). Mean ± SD, ^*^ *p* < 0.05, ^**^ *p* < 0.01, ^***^ *p* < 0.001 indicate significant differences, and *ns* > 0.05 means no significance difference.

KD mice exhibited symptoms including pyrexia, body mass reduction, vascular inflammation, and splenomegaly (Kang and Kim, 2019). Therefore, the mouse spleens from each group were observed (Figure 2A), and the weights of both the spleen and body in each group were measured. The results showed that spleen enlargement in CAWS-induced KD mice was improved after FA treatment, but there was no change in body weight. Additionally, the ratio of spleen weight to body weight in CAWS-induced KD mice was also reduced, but this ratio in either CAWS + L-FA or CAWS + H-FA group were significantly higher than that of control group (Figure 2B). Furthermore, we obtained protein and mRNA from the aortic root and proximal coronary artery regions of mouse heart tissues in each group for subsequent RT-qPCR or western blot analyses. RT-qPCR results demonstrated that FA administration could reduce the mRNA expression levels of inflammatory cytokines such as *TNF-α*, *IL-1β*, and *IL-6* as well as chemokines including *CCL2*, *CCL7*, *CCL17*, *CXCL5*, and *CXCL10* in CAWS-induced KD mice, but the expression levels of these inflammatory cytokines and chemokines in either CAWS + L-FA or CAWS + H-FA group were almost significantly higher than these of control group (Figure 2C). In addition, the expressions of CD45, IL-1β, and IL-6 were examined by western blot assay (Figure 2D). The results indicated that compared with control group, the levels of these inflammatory cytokines were markedly upregulated in CAWS-induced KD mice, but either L-FA or H-FA significantly inhibit these inflammatory cytokine expressions. Although there was almost no significant difference between control and L-FA or H-FA treatment, but the levels of these inflammatory cytokines in either CAWS + L-FA or CAWS + H-FA group were higher than these of control group (Figure 2E). These data further suggest that FA can alleviate inflammation in CAWS-induced KD mice, but FA alone is hard to completely restore KD mice into normal condition.

### FA inhibited vascular endothelial cell apoptosis of coronary arteries in CAWS-KD mice

**FIGURE 3:**
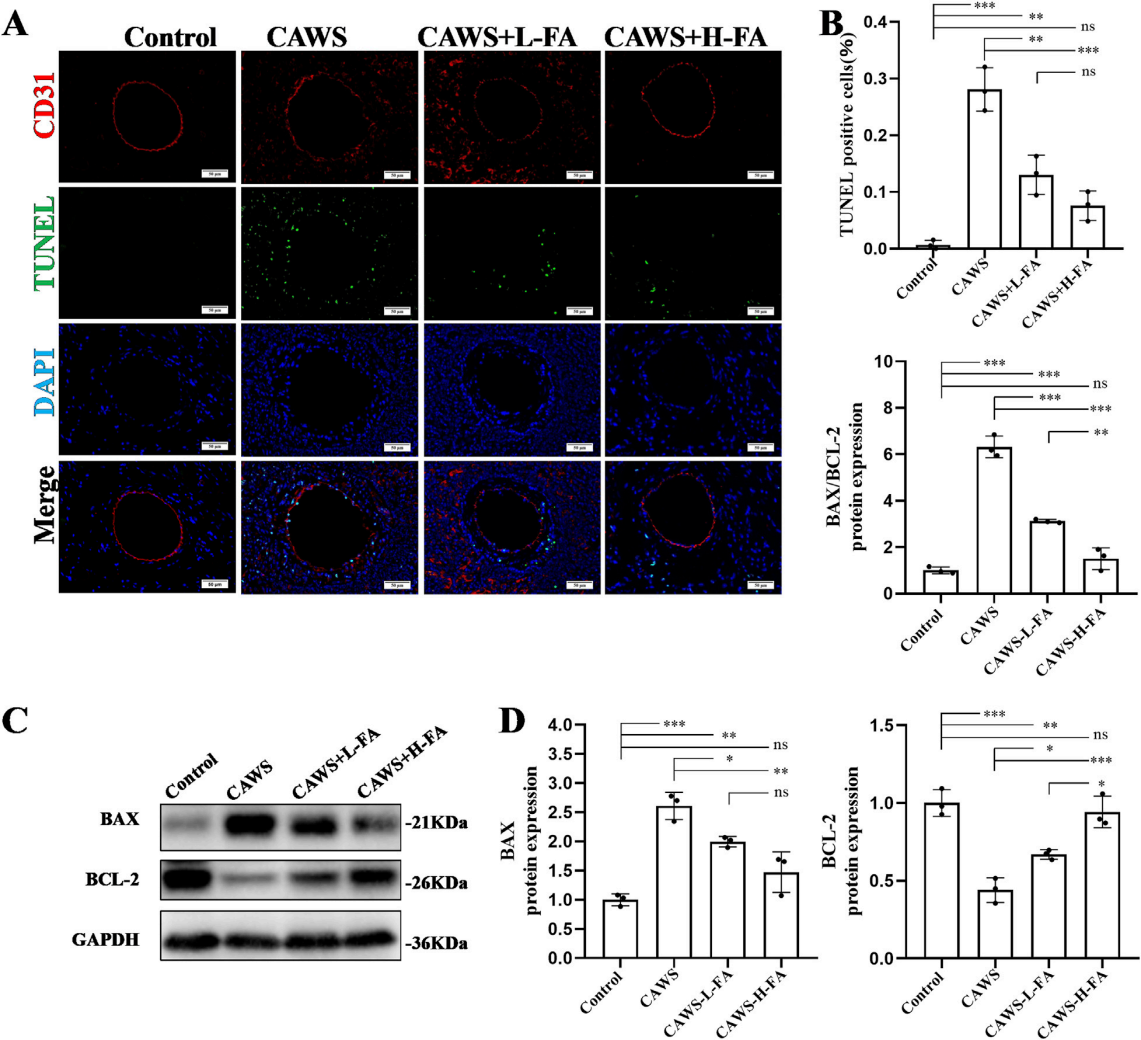
FA inhibited vascular endothelial cell apoptosis of coronary arteries in CAWS-KD mice. **(A)** TUNEL and CD31 staining of paraffin sections in different groups. Scale bars 50 μm. **(B)** The quantification of TUNEL-positive vascular endothelial cells in different groups (n = 3). **(C)** The expression levels of BAX and BCL-2 proteins in cardiac tissues of mice in different experimental groups were evaluated using Western blot analysis. **(D)** The quantification of BAX and BCL-2 expressions in different groups (n = 3). Mean ± SD, ^*^ *p* < 0.05, ^**^ *p* < 0.01, ^***^ *p* < 0.001 indicate significant differences, and *ns* > 0.05 means no significance difference.

To assess the anti-apoptotic effect of FA on the coronary endothelial cells in KD mice, CD31/TUNEL double staining was performed (Figure 3A). The percentage of TUNEL-positive cells in CD31-positive coronary endothelial cells was high in CAWS-induced KD mice, and FA treatment significantly reversed this trend, but the TUNEL-positive cells in CD31-positive coronary endothelial cells of either CAWS + L-FA or CAWS + H-FA group were higher than these of control group (Figure 3B). In addition, the expressions of apoptosis protein BAX and anti-apoptosis protein BCL-2 were detected by werstern blot analysis (Figure 3C). The quantitative results showed that compared with control group, the level of apoptosis protein BAX was significantly upregulated, while the level of anti-apoptosis protein BCL-2 as well as the ratio of BAX/BCL-2 were significantly downregulated in CAWS group. Either L-FA or H-FA treatment could reverse these trends compared with CAWA group but not control group (Figure 3D). These results suggest that FA can improve the apoptosis of coronary arteries in CAWS-induced KD mice, but FA alone is hard to completely restore KD mice into normal condition.

### FA had protective effects on CAWS-KD mice through activating the AMPK/mTOR/NF-κB pathway

**FIGURE 4:**
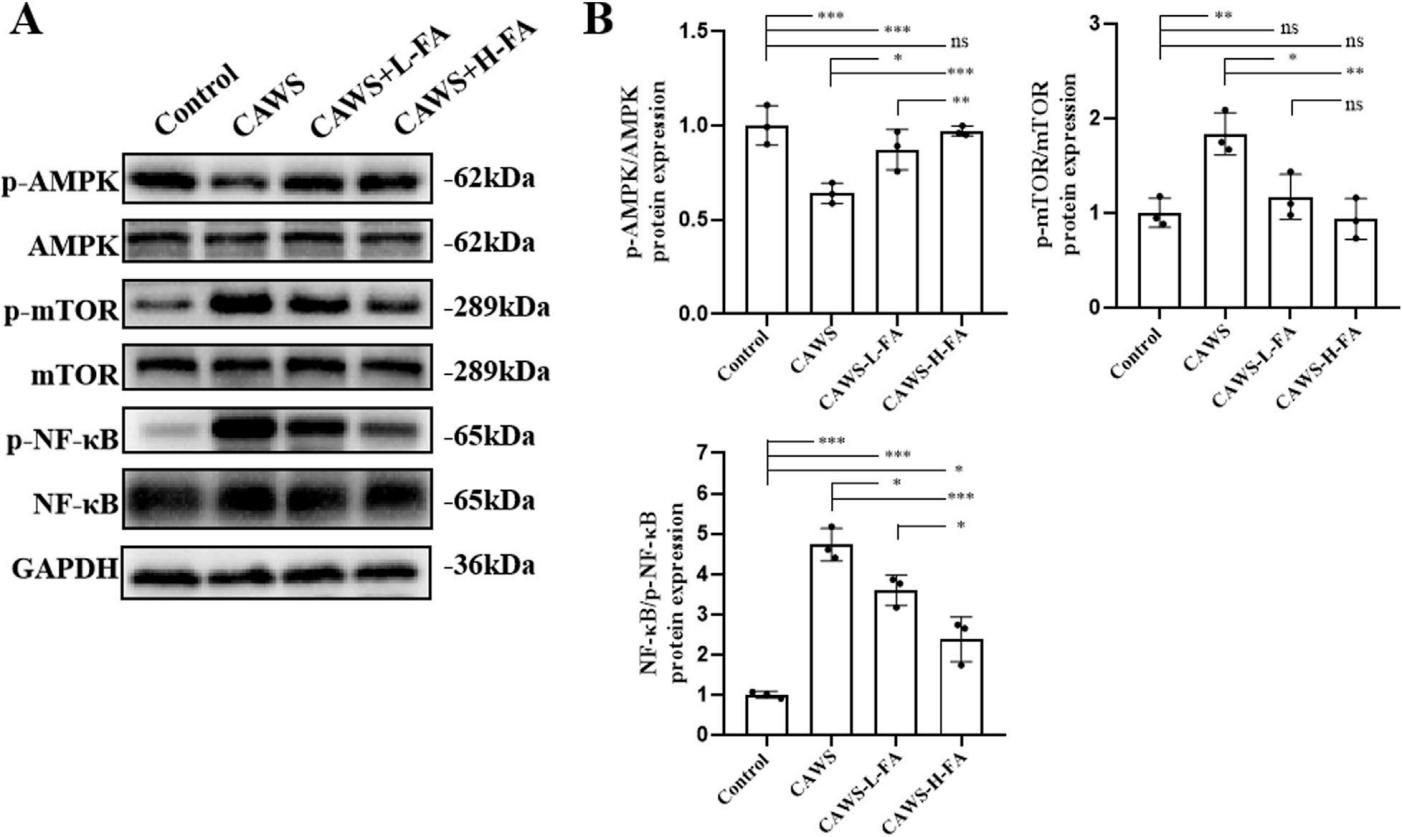
FA had protective effects on CAWS-KD mice through activating AMPK/mTOR/NF-κB pathway. **(A)** Western blot analysis was employed to assess the expression of relevant biomarkers along the AMPK/mTOR/NF-κB pathway in the aortic root and proximal coronary artery regions of mouse heart tissues in different groups. **(B)** The statistical analysis of western blot in different groups (n = 3). Mean ± SD, ^*^ *p* < 0.05, ^**^ *p* < 0.01, ^***^ *p* < 0.001 indicate significant differences, and *ns* > 0.05 means no significance difference.

Previous studies have shown that overactivation of the NF-κB pathway is closely related to the occurrence of coronary artery damage in KD (Ichiyama et al., 2001). Then, we obtained protein from the aortic root and proximal coronary artery regions of mouse heart tissues in each group for subsequent western blot analyse. Activation of AMPK has an antagonistic effect on mTOR, and NF-κB is downstream of mTOR. Therefore, the AMPK/MTOR-mediated NF-κB pathway was detected by western blot to assess the protective effects of FA on KD mice (Figure 4A). The results showed that p-AMPK/AMPK levels were downregulated but p-mTOR/mTOR and p-NF-κB/NF-κB levels were upregulated in CAWS group compared with control group. Both L-FA and H-FA treatments could reverse these trends compared with CAWA group but not control group (Figure 4B). These results suggest that FA may exert partially protective effects on CAWS-induced KD mice through activating the AMPK/mTOR/NF-κB pathway.

### FA alleviated cell inflammation of TNF-α-induced HUVECs through inhibiting the NF-κB pathway

The TNF-α-induced HUVECs were used as a KD cell model *in vitro*. The optimum working concentration of TNF-α was explore using CCK-8 assay. We set different concentrations of TNF-α ranging from 0.001 ng/mL to 1,000 ng/mL to treat HUVECs. The results showed that the cell viability of HUVECs could be greatly inhibited by 1,000 ng/mL TNF-α. Therefore, we chose 1 μg/mL as the working concentration of TNF-α (Supplementary Figure S1A). The treatment of FA at various concentrations did not exert a significant impact on cell viability, indicating that FA has no potential cytotoxic effect on HUVECs (Supplementary Figure S1B). In addition, the cell activity of TNF-α-induced HUVECs was significantly improved with 20 μM FA intervention, which was selected as the working concentration (Supplementary Figure S1C).

**FIGURE 5:**
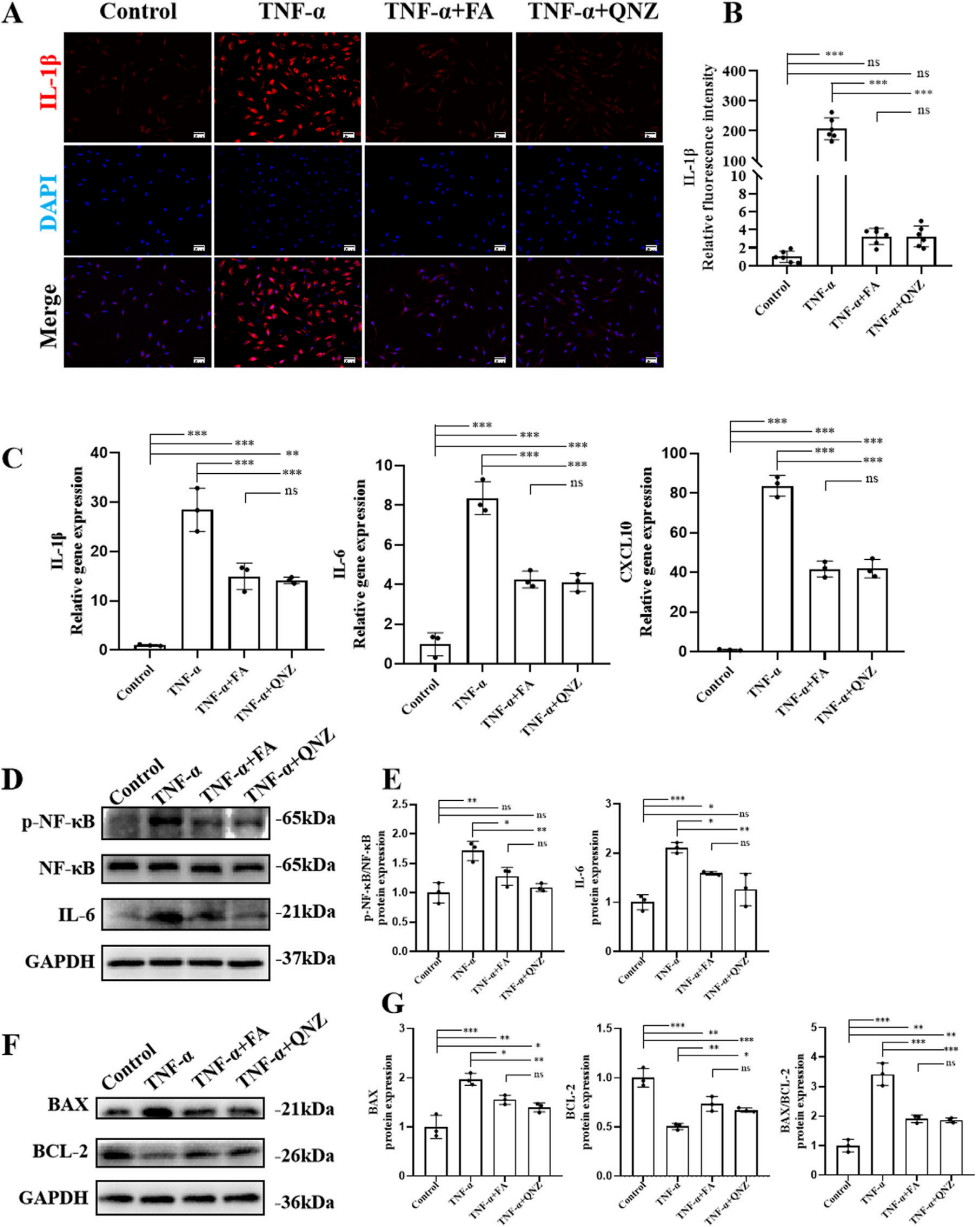
FA alleviated cell inflammation of TNF-α-induced HUVECs through inhibiting NF-κB pathway. **(A)** Immunofluorescence staining of IL-1β in each group. Scale bars 50 μm. **(B)** The quantification of protein levels in each group (n = 3). **(C)** The gene expression levels of inflammatory cytokines such as IL-1β and IL-6 as well as chemokine CXCL10 in different groups by RT-qPCR (n = 3). **(D)** The expressions of proteins such as NF-κB, p-NF-κB, IL-1β, and IL-6 in different groups by western blot. **(E)** The statistical analysis of western blot gel bands in different groups (n = 3). **(F)** The protein expressions of BAX and BCL-2 in different groups by western blot. **(G)** The quantification of western blot in different groups (n = 3). Mean ± SD, ^*^ *p* < 0.05, ^**^ *p* < 0.01, ^***^ *p* < 0.001 indicate significant differences, and *ns* > 0.05 means no significance difference.

The anti-inflammatory effect of FA on TNF-α-induced HUVECs was investigated. The result of p-NF-κB immunofluorescence staining (Supplementary Figure S2A) showed that TNF-α induction increased the level of p-NF-κB in the nucleus of HUVECs, and FA effectively reduce the p-NF-κB level, while the NF-κB inhibitor QNZ could also inhibit p-NF-κB level in TNF-α-induced HUVECs (Supplementary Figure S2B). In addition, the results of IL-1β immunofluorescence staining (Figure 5A) showed that the trend of IL-1β expression in each group was consistent with that of p-NF-κB immunofluorescence staining result (Figure 5B). Furthermore, we obtained protein and mRNA from HUVECs in each group for subsequent RT-qPCR or western blot analyses. FA treatment could also significantly inhibit the gene expressions of inflammatory cytokines, such as *IL-1β*, *IL-6*, and chemokine *CXCL10 in* TNF-α-induced HUVECs, which was similar to NF-κB inhibitor QNZ adminstration, but the expression levels of these inflammatory cytokines and chemokine in either TNF-α + FA or TNF-α + QNZ group were significantly higher than these of control group (Figure 5C). In addition, the protein expressions of NF-κB, p-NF-κB, and IL-6 were detected in each group by western blot (Figure 5D), showing a similar trend to RT-qPCR result that either FA or QNZ could downregulate these protein expressions in TNF-α-induced HUVECs (Figure 5E). Moreover, the apoptosis protein BAX and anti-apoptosis protein BCL-2 expression were also analyzed by western blot (Figure 5F), indicating that compared with control group, the level of BAX was significantly upregulated, and the level of BCL-2 as well as the ratio of BAX/BCL-2 were significantly downregulated in TNF-α group, which could be reversed by treatment with either FA or QNZ compared with TNF-α group but not control group (Figure 5G). These findings suggest that FA can reduce TNF-α-induced inflammation in HUVECs through inhibiting the NF-κB pathway, but FA or QNZ alone is hard to completely restore KD HUVECs into normal condition.

### FA alleviated cell inflammation of TNF-α-induced HUVECs

**FIGURE 6:**
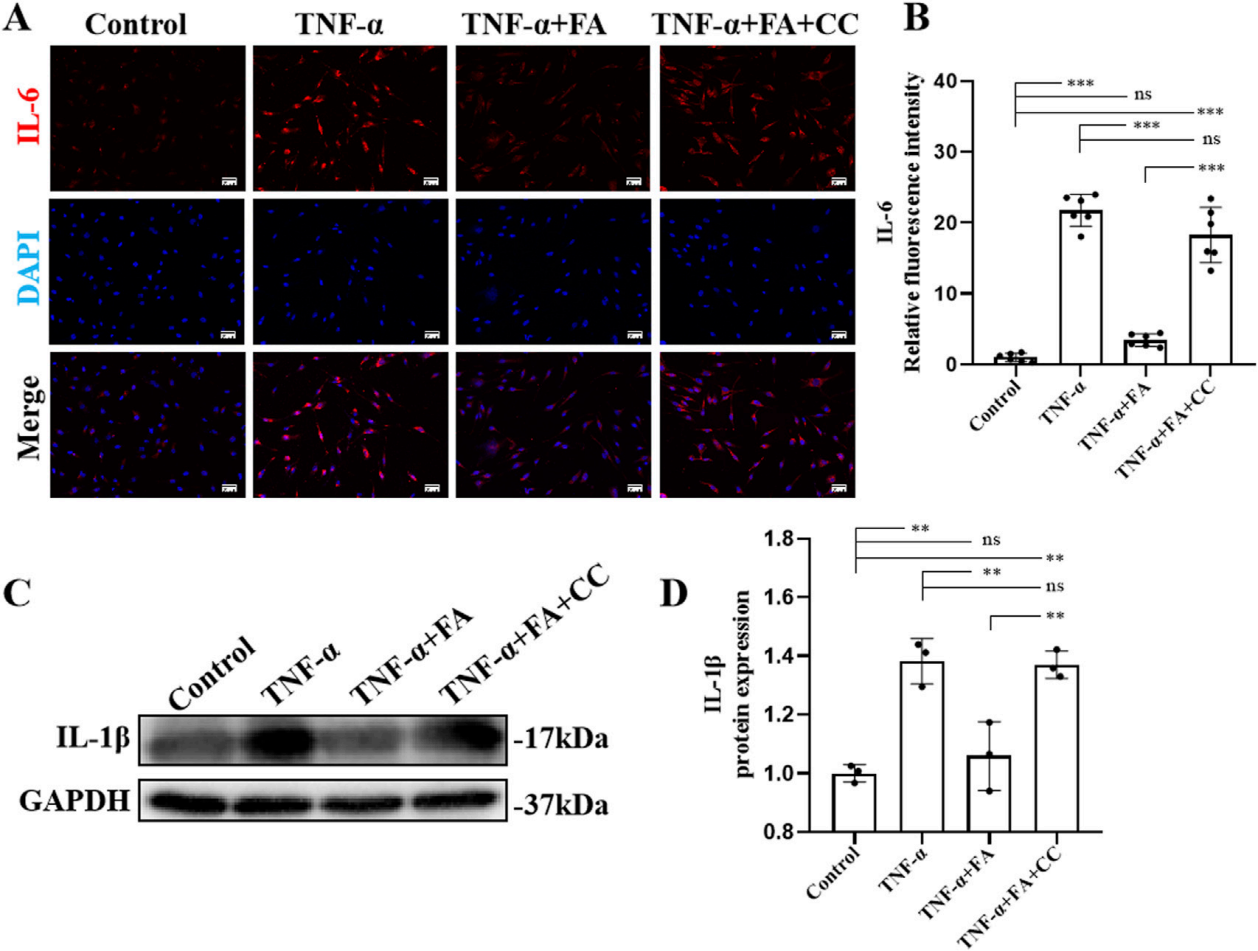
FA alleviated cell inflammation of TNF-α-induced HUVECs. **(A)** Immunofluorescence staining of IL-6 in different groups. Scale bars 50 μm. **(B)** The statistical analysis of IL-6 fluorescence intensity in different groups (n = 3). **(C)** Western blot gel images of IL-1β. **(D)** The bar graphs represent the quantification of protein levels in each group (n = 3). Mean ± SD, ^*^ *p* < 0.05, ^**^ *p* < 0.01, ^***^ *p* < 0.001 indicate significant differences, and *ns* > 0.05 means no significance difference.

The AMPK inhibitor compound (CC) was also used in this study, in addition to QNZ, to further explore the mechanism of FA’s protective effect on HUVECs. The results of IL6 immunofluorescence staining (Figure 6A) showed that FA could reduce IL6 expression, and CC could significantly reverse the effect of FA compared with TNF-α group but not control group (Figure 6B). Additionally, western blot analysis was performed to detect the expression of IL-1β protein in different groups (Figure 6C). The results showed that IL-1β levels were upregulated in TNF-α group compared with control group, and this trend was reversed by FA treatment, but CC almost neutralized the effect of FA compared with TNF-α group but not control group (Figure 6D). These results suggest that FA can reduce TNF-α-induced HUVECs inflammation by activating AMPK.

### FA alleviated cell apoptosis of TNF-α-induced HUVECs

**FIGURE 7:**
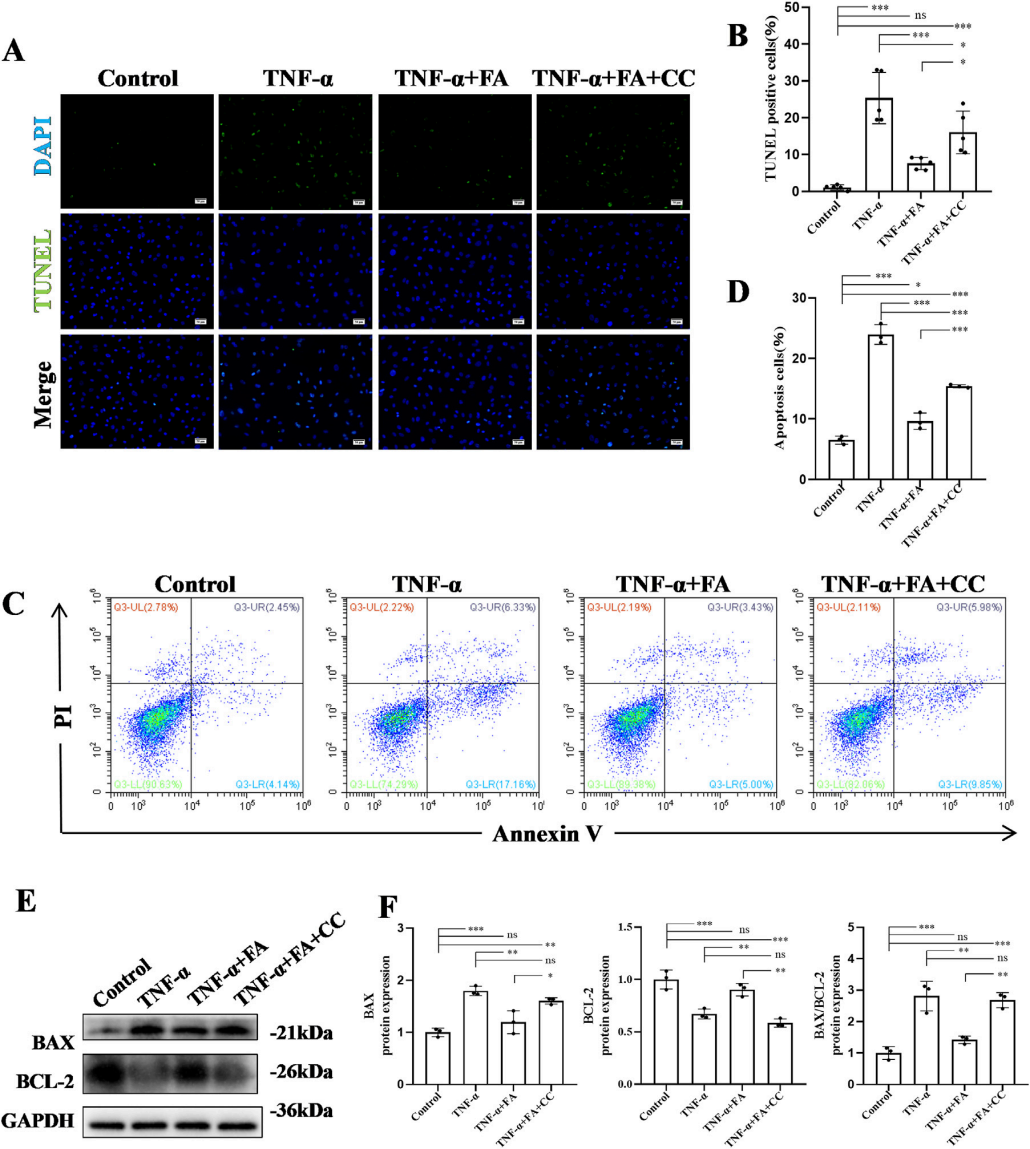
FA alleviated cell apoptosis of TNF-α-induced HUVECs. **(A)** TUNEL staining of HUVECs in different groups. Scale bars 50 μm. **(B)** The statistical analysis of TUNEL-positive cells in different groups (n = 3). **(C)** The apoptosis of HUVECs in different groups by flow cytometry. **(D)** The statistical analysis of flow cytometry in different groups (n = 3). **(E)** The protein expressions of BAX and BCL-2 in different groups using western blot. **(F)** The statistical analysis of BAX and BCL-2 levels in different groups (n = 3). Mean ± SD, ^*^ *p* < 0.05, ^**^ *p* < 0.01, ^***^ *p* < 0.001 indicate significant differences, and *ns* > 0.05 means no significance difference.

To further investigate the anti-apoptosis effect of FA on TNF-α-induced HUVECs *in vitro*, TUNEL staining was conducted in each group (Figure 7A). The results of TUNEL staining showed a significant increase in the number of TUNEL-positive cells in TNF-α group compared with control group, while FA treatment decreased the numbers of TUNEL-positive cells, but this anti-apoptosis effect could be neutralized by the AMPK inhibitor CC compared with TNF-α group but not control group (Figure 7B). Flow cytometry with Annexin V-PI was also performed to detect the ratio of apoptosis in each group (Figure 7C). The result indicated that the percentage of apoptotic cells in FA treatment group was significantly decreased from 23.94% to 10.04%, but CC treatment counteracted the effect of FA compared with TNF-α group but not control group (Figure 7D). In addition, western blot analysis was conducted to detect the expressions of apoptotic protein BAX and anti-apoptotic protein BCL-2 in each group (Figure 7E), and the result trend was consistent with the above flow cytometry result (Figure 7F). These data indicate that FA can alleviate TNF-α-induced apoptosis in HUVECs, but FA alone is hard to completely restore KD HUVECs into normal condition.

### FA exerted protective effects on TNF-α-induced HUVECs through activating the AMPK/mTOR/NF-κB pathway

**FIGURE 8:**
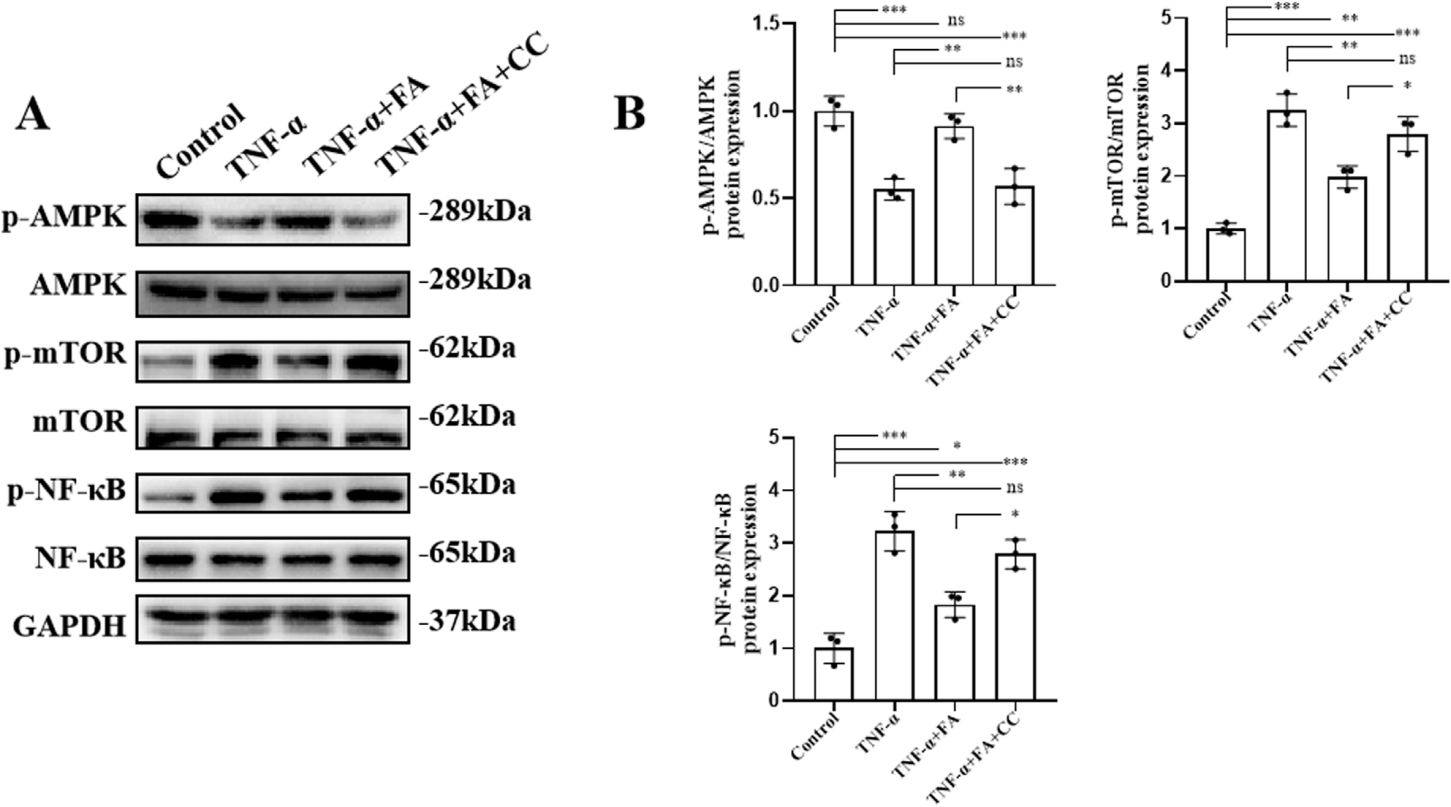
FA exerted protective effects on TNF-α-induced HUVECs through activating AMPK/mTOR/NF-κB pathway. **(A)** The protein expressions of the associated indicators of AMPK/mTOR/NF-κB pathway in different groups by western blot. **(B)** The quantification of western blot in different groups (n = 3). Mean ± SD, ^*^ *p* < 0.05, ^**^ *p* < 0.01, ^***^ *p* < 0.001 indicate significant differences, and *ns* > 0.05 means no significance difference.

**FIGURE 9:**
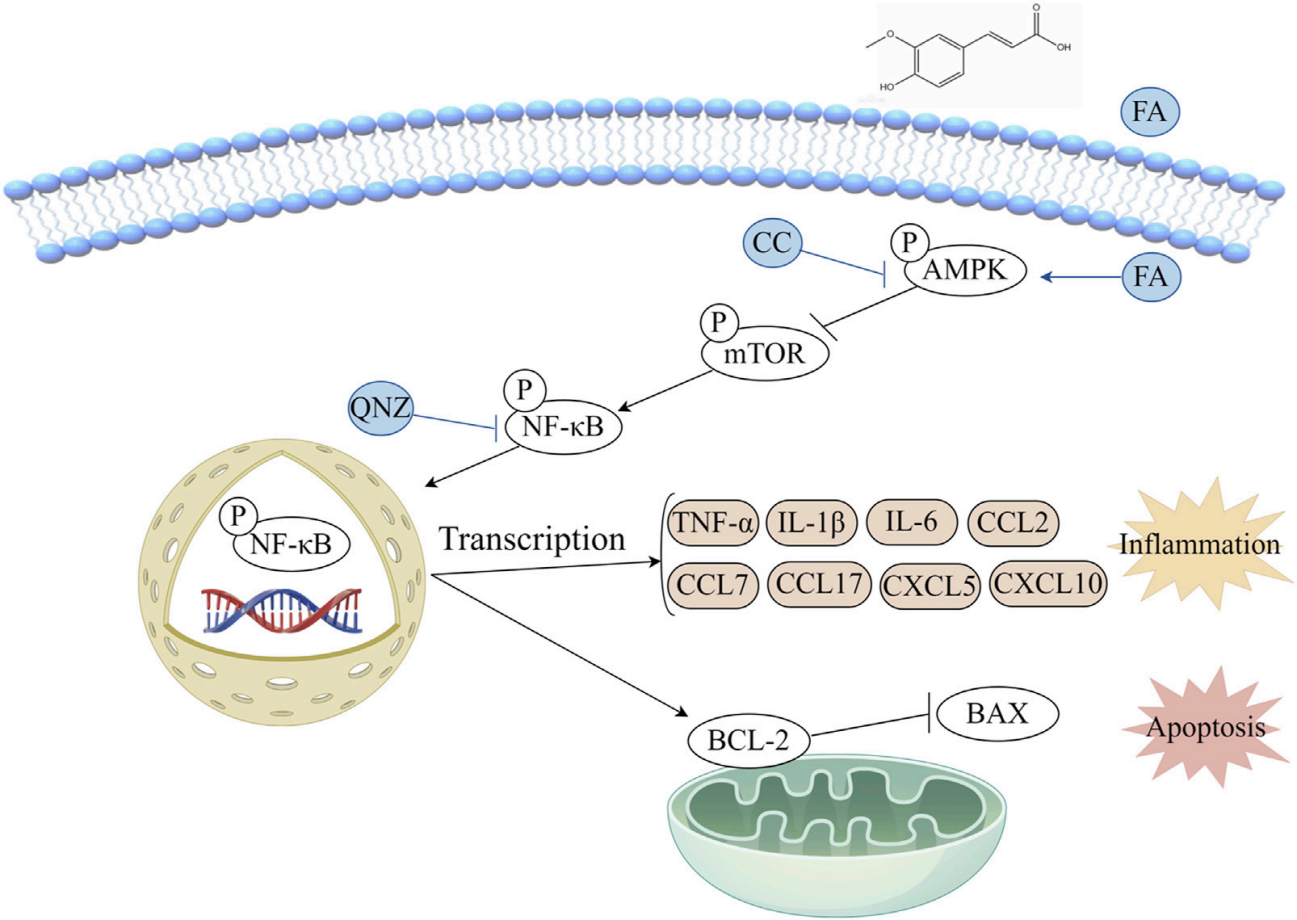
Diagram of the potential mechanism involved in the protective effects of FA on KD.

In order to explore the potential mechanism of FA effect *in vitro*, the AMPK/mTOR-mediated NF-κB pathway was analyzed by western blot (Figure 8A). The results showed that compared with control group, the level of p-AMPK/AMPK was downregulated, and the levels of p-mTOR/mTOR and p-NF-κB/NF-κB were significantly upregulated in TNF-α group. FA treatment significantly alleviated these trends, but the AMPK inhibitor CC neutralized the effect of FA compared with TNF-α group but not control group (Figure 8B). Based on the above findings, we believe that FA can exert the partially protective effects on KD through activating the AMPK/mTOR/NF-κB pathway involved in anti-inflammation and anti-apoptosis (Figure 9), but FA alone is hard to completely restore KD mice or HUVECs into normal condition.

## Discussion

KD is an acute systemic vasculitis, and its etiology and pathogenesis are still unclear. Currently, IVIG treatment works well in the majority of KD patients, but more than 20% of KD patients exhibit IVIG tolerance and need adjuvant drug therapy (McCrindle et al., 2017). Therefore, it is necessary for us to develop more strategies for KD adjuvant therapy combined with the current treatment regimen. In this study, CAWS-induced KD mouse model *in vivo* was established, and we confirmed the partially protective effects of FA on KD mice by alleviating coronary artery inflammation and inhibiting vascular endothelial cell apoptosis through activating the AMPK/mTOR/NF-κB pathway. Moreover, the TNF-α-induced KD HUVEC model *in vitro* was also established, and we also demonstrated the partially protective effect of FA on KD HUVECs by alleviating cell inflammation and reducing cell apoptosis through activating the AMPK/MTOR/NF-κB pathway. However, FA alone is hard to completely restore KD into normal condition in our study. Therefore, the further researches on FA as an adjuvant for the current KD therapy should be conducted.

Natural products and their derivatives have been the focus of research and development for novel pharmaceuticals. FA is mainly found in medicinal herbs, such as Angelica sinensis, Diels, and Ligusticum chuanxiong Hort (Wang et al., 2015). FA is also found in staple foods, fruits, and vegetables, and has been used as an additive in food, pharmaceuticals and cosmetic preparations due to its antioxidant properties (Graf, 1992). The structural form of FA is 3-(4-hydroxy-3-methoxyphenyl)-2- propenoic acid, and FA has two conformation of trans-FA and cis-FA, which exist in liquid and solid states, respectively. The stability of trans-FA surpasses that of cis-FA and the polypharmacological properties of both almost have no difference, but trans-FA is more popular employed (Babbar et al., 2021; Sanshita et al., 2024). So, we also utilized trans-FA in this study. FA is a polypharmacologically active phenolic acid whose antioxidant capacity depends on its structural characteristics, especially the electron-donating groups in the benzene ring, which give FA the ability to scour free radicals (Graf, 1992). Sodium ferulate is a sodium salt derived from FA, which is widely used in the treatment of cardiovascular diseases and the prevention of thrombosis (Wang and Ou-Yang, 2005). FA also has strong anti-inflammatory effect by inhibiting the expression of related inflammatory factors through multiple molecular pathways, such as nuclear factor kappa-B (NF-κB) and cell adhesion molecules (CAM) (Li et al., 2021). The anti-inflammatory effect of FA contributed its application in many diseases, such as neurotoxicity (Liu et al., 2017), liver dysfunction (Roghani et al., 2020), kidney damage (Jung et al., 2009) and heart dysfunction (Neto-Neves et al., 2021). FA also mediates the anti-apoptosis effect through downregulating BAX and upregulating BCL-2 expression by inhibiting the JNK pathway (Li et al., 2021).

The abnormality of coronary arteries in KD patients is associated with the disturbance of vascular endothelial cell homeostasis (Ueno et al., 2017). Vascular endothelial cells play an important role in maintaining the function of blood vessels. There are many pathological mechanisms of vascular endothelial cell injury in KD, such as cellular inflammation, apoptosis, pyroptosis, and endothelial-to-mesenchymal transition (EndoMT) (He et al., 2017; Jia et al., 2019; Wu et al., 2018). KD is associated with infiltration of multiple innate and adaptive immune cells into the coronary artery wall, including monocytes, macrophages, and neutrophils in the artery wall as well as the accumulation of activated CD8 T cells (cytotoxic T lymphocytes) and IgA plasma cells (Orenstein et al., 2012). CD45 (Lymphocyte Common antigen) is a receptor linker protein tyrosine phosphatase, which is expressed on all leukocytes and plays a crucial role in antigen receptor signal transduction, lymphocyte development and other cellular functions (Hermiston et al., 2003). Hence, we conducted CD45 immunohistochemical staining to assess the recruitment of inflammatory cells, and our results showed that FA could ameliorate coronary artery leukocyte aggregation in CAWS-induced KD mice, but FA alone was hard to completely alleviate the inflammatory of KD mice into normal condition. The inflammatory response of KD is not only obvious in the heart, but also in the spleen. The spleen serves as an important organ for peripheral inflammation and is often characterized by splenomegaly due to the release of lymphocytes, monocytes, NK cells, and neutrophils from the spleen (Monsour and Borlongan, 2023). Splenomegaly is also observed in clinical KD and is associated with an overactivated inflammatory response (Kang and Kim, 2019). In our study, FA treatment significantly ameliorated splenomegaly in CAWS-induced mice, but FA alone was hard to completely meliorate splenomegaly of KD mice into normal condition.

Immune cells can release pro-inflammatory cytokines, such as TNF and IL-1β, to promote vascular endothelial cell damage (Leung et al., 1986). Single-cell sequencing data of KD patients have confirmed that pro-inflammatory genes, such as *IL-1β* and *TNF-α*, were significantly upregulated (Wang et al., 2021). Therefore, reducing the levels of inflammatory factors is considered as a therapeutic strategy for KD. The TNF inhibitor Etanercep and the IL-1 inhibitor Anabusin have been clinically used to treat KD (Tacke et al., 2012). IL-1β, TNF-α, and IL-6 can stimulate endothelial cells and leukocytes to produce a series of chemokines, such as CCL2, CCL7, CCL17, CXCL5, and CXCL10, which can recruit inflammatory cells to the coronary artery site, and these chemokines are also elevated in KD mouse model (Feng et al., 2015; Hosaka et al., 2024; Stock et al., 2016). Therefore, in this study, we also detected the expression levels of pro-inflammatory cytokines, such as IL-1β, TNF-α, and IL-6 as well as chemokines, such as CCL2, CCL7, CCL17, CXCL5, and CXCL10, and found that FA effectively downregulated these factor expressions, but FA alone was hard to completely inhibit these factor expressions of KD mice into normal condition.

In addition to inflammation, endothelial cell apoptosis is also a key pathological mechanism of KD (Jiang et al., 2016; Wu et al., 2018). The biochemical marker of early and late apoptotic cells is the cleavage of double-stranded genomic DNA by endonuclidenase, exposesing the free 3′-hydroxyl terminus, and TUNEL staining can be used to detect DNA breaks by labeling the free 3′-hydroxyl terminus (Mirzayans and Murray, 2020). Therefore, we performed TUNEL staining to detect cell apoptosis in each group, our results showed that FA treatment could significantly decrease the percentage of TUNEL-positive cells in CD31-positive coronary endothelial cells of CAWS-induced KD mice. In addition, Annexin V and PI staining can also be used to assess cell apoptosis based on flow cytometry. Annexin V-negative and PI-negative were non-apoptotic cells. Annexin V-positive and PI-negative were early apoptotic cells. Annexin V-positive and PI-positive were late apoptotic or necrotic cells. Annexin V-negative and PI-positive were dead cells (Chen et al., 2008). In this study, FA could significantly reduce the percentage of early and late apoptotic cells in TNF-α-induced HUVECs. However, FA alone was hard to completely alleviate cell apoptosis of KD mice and HUVECs into normal condition.

Previous studies have reported that the NF-κB pathway was activated in KD patients, which played an important role in the inflammatory response (Ichiyama et al., 2001; Tian et al., 2017). NF-κB is a transcription factor that play an important role in a variety of physiological and pathological processes (Yu et al., 2020). The main transcriptional active form of NF-κB is p65/p50 heterodimer, with IκBα being the most common inhibitor. Phosphorylation of IκBα can cause the release of NF-κB from the complex. Translocation of free NF-κB dimer into nuclear NF-κB can facilitate transcription to regulate the expression of pro-inflammatory cytokines (IL-1β, IL-6, and TNF-a), chemokines, and adhesion molecules (Perkins, 2007). FA could inhibit the activation of NF-κB to regulate the expression of inflammatory factors in elderly kidneys (Jung et al., 2009). FA could also significantly attenuate the production of TNF-α, IL-1β, and IL-6 through inhibiting the NF-κB pathway (Liu et al., 2017). In our study, we also demonstrated that FA effectively attenuated NF-κB phosphorylation and its subsequent translocation into the nucleus, exerting an anti-inflammatory effect, but FA alone was hard to completely attenuate inflammation of KD into normal condition.

Cell apoptosis is a mechanism of programmed cell death. BCL-2 family proteins mediate mitochondrial apoptosis by regulating mitochondrial outer membrane permeability. BCL-2 family proteins include anti-apoptotic protein BCL-2 and pro-apoptotic protein BAX (Volkmann et al., 2014). BAX protein is mostly distributed in the cytoplasm in the form of inactive monomer. Only after receiving the apoptotic signal and being activated, the molecular conformation is changed, translocated, and inserted into the mitochondrial outer membrane to form Bax/Bax homologous dimer protein channels, which destroy the integrity of mitochondrial membrane and promote the release of cytochrome c (Cyt-c) (Spitz and Gavathiotis, 2022). The overexpressed BCL-2 protein can form heterodimer with BAX to inhibit the translocation and dimerization of BAX, block the release of Cyt-c, and effectively inhibit the occurrence of apoptosis (Park and Hockenbery, 1996). FA could attenuate TNF-α level, upregulate BCL-2 expression, downregulate BAX expression, and reduce hepatocyte apoptosis induced by ischemia/reperfusion (IR) (Kim and Lee, 2012). FA could also improve placental apoptosis in NG-nitro-l-arginine methyl ester (L-NAME)-induced preeclampsia (PE) rat model through increasing BCL-2 expression and decreasing BAX expression (Chen et al., 2019). NF-κB is involved in pro-apoptotic processes (Kühnel et al., 2000). The activation of NF-κB pathway plays an important role in TNF-α induced cardiomyocyte apoptosis of cardiomyocytes (Dhingra et al., 2009). In this study, after TNF-α treatment, the phosphorylation level of NF-κB was indeed increased, and the ratio of BAX/BCL-2 was upregulated, suggesting that TNF-α-induced NF-κB activation may promote apoptosis of endothelial cells. Subsequently, FA like NF-κB inhibitor QNZ could attenuate this change, suggesting that FA may inhibit apoptosis through inhibiting NF-κB activation.

The adenosine monophosphate-activated protein kinase (AMPK) is a central regulator of cellular energy metabolism in eukaryotes, and the phosphorylation of AMPKα at Thr172 is required for AMPK activation (Hawley et al., 1996). mTOR kinase is involved in the regulation of many biological processes, such as energy metabolism, autophagy, and inflammation. mTOR kinase is present in two different multiprotein complexes mTOR Complex 1 (mTORC1) and mTOR Complex 2 (mTORC2) (Laplante and Sabatini, 2012). mTOR is a downstream signaling effector of AMPK pathway. AMPK can inhibit mTOR signaling through direct phosphorylation of TSC2 (the tumor suppressor) and Raptor (a protein associated with mTOR regulation) (Mihaylova and Shaw, 2011). It has been reported that FA possessed the ability to suppress LPS-induced neuroinflammation of BV2 microglia through activating the AMPK/mTOR pathway (Chen et al., 2023). The NF-κB pathway is downstream of mTOR, and mTOR-mediated IKK activation could lead to the activation of NF-κB (Ahmad et al., 2013; Dan et al., 2008). Previous study has demonstrated that the inhibition of mTOR/NF-κB pathway could ameliorate neuroinflammation, synaptic damage, and other symptoms associated with diabetic encephalopathy (Xu et al., 2021). Senescent cells under hypoxia conditions could cause AMPK activation, followed by AMPK-mediated inhibition of the mTOR/NF-κB pathway (van Vliet et al., 2021). In this study, FA treatment could significantly increase the level of phosphorylated AMPK, decrease the level of phosphorylated mTOR, downregulate the expression of BAX, and upregulate the level of BCL-2 in CAWS induced KD mice and TNF-α-induced HUVECs.

Compound C is a reversible and selective inhibitor of AMPK with a Ki of 109 nM (Zhou et al., 2001). QNZ (EVP4593), a cell permeable quinazoline compound, is an inhibitor of the NF-κB pathway, and can inhibit NF-κB transcriptional activation with an IC50 of 11 nM (Xu and Russu, 2013). Compound C and QNZ inhibitors can modulate the AMPK/mTOR/NF-κB pathway. In this study, Compound C could reverses the therapeutic effect of FA on TNF-α-induced HUVECs through inhibiting the AMPK/mTOR pathway, while QNZ exerted an anti-inflammatory role similar to FA by inhibiting the NF-κB signaling.

There are still certain limitations in our present study that we just confirmed the therapeutic effects of FA on CAWS-induced KD mouse model. Although previous studies have reported that there are certain histopathological similarities in the aortic root and proximal coronary artery region between CAWS-induced KD mouse model and KD patients, there are also some differences, for instance inflammation also affects non-coronary artery sites (Noval Rivas and Arditi, 2020). The established KD endothelial cell model is best induced by serum samples of KD patients (Jia et al., 2021), which are very precious and difficult for us to obtain in this study. Moreover, FA combination with the current treatment regimen for KD was not investigated.

In conclusion, this study demonstrated that FA could exert anti-inflammatory and anti-apoptotic effects on KD mice and HUVECs through activating the AMPK/mTOR/NF-κB pathway, but FA alone is hard to completely restore KD into normal condition. Therefore, FA as an adjuvant to the current KD treatment regimen should be further explored.

## Data Availability

The raw data supporting the conclusions of this article will be made available by the authors, without undue reservation.
