# Supplementary figures and images for: Ferulic acid suppresses the inflammation and apoptosis in Kawasaki disease through activating the AMPK/mTOR/NF-κB pathway
Source: Front Pharmacol. 2024 Aug 29;15:1420602. doi: 10.3389/fphar.2024.1420602 (PMC11390509; doi:10.3389/fphar.2024.1420602)

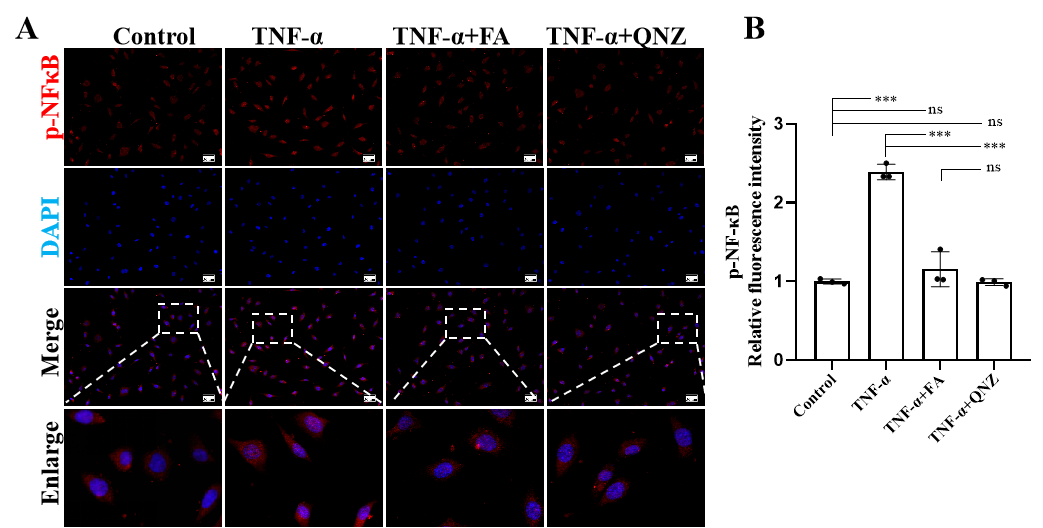

Supplement: Supplementary file 1 [file Image2.tif]

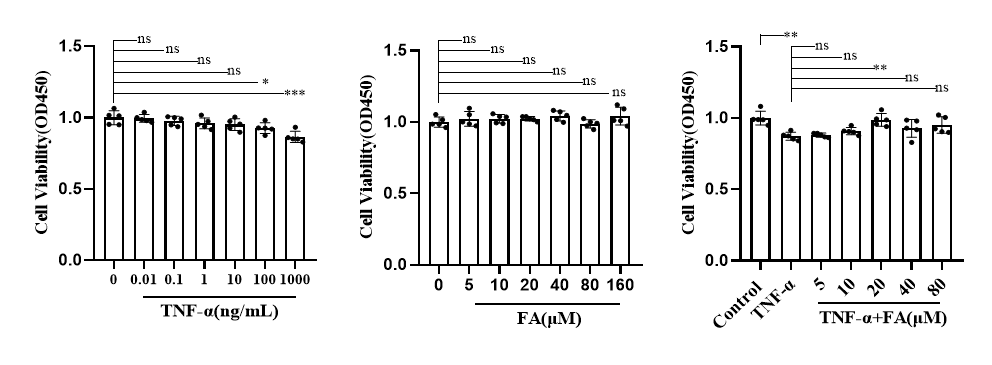

Supplement: Supplementary file 2 [file Image1.tif]
